# mRNA m5C Alteration in Azacitidine Demethylation Treatment of Acute Myeloid Leukemia

**DOI:** 10.1002/mc.23864

**Published:** 2024-12-17

**Authors:** Ziwei Chen, Yingyu Guo, Zaifeng Zhang, Chang Li, Lili Zhang, Ye Liu, Gaoyuan Sun, Fei Xiao, Ru Feng, Chunli Zhang

**Affiliations:** ^1^ The Key Laboratory of Geriatrics, Beijing Institute of Geriatrics, Institute of Geriatric Medicine Chinese Academy of Medical Sciences, Beijing Hospital/National Center of Gerontology of National Health Commission Beijing China; ^2^ Graduate School of Peking Union Medical College Chinese Academy of Medical Sciences Beijing China; ^3^ Clinical Biobank, Beijing Hospital, National Center of Gerontology, National Health Commission, Institute of Geriatric Medicine Chinese Academy of Medical Sciences Beijing China; ^4^ Center of Laboratory Medicine, National Clinical Research Center of Cardiovascular Diseases, Fuwai Hospital Chinese Academy of Medical Sciences & Peking Union Medical College/National Center for Cardiovascular Diseases Beijing China; ^5^ Department of Hematology, Beijing Hospital, National Center of Gerontology, National Health Commission, Institute of Geriatric Medicine Chinese Academy of Medical Sciences Beijing China

**Keywords:** acute myeloid leukemia, nanopore sequencing, RNA 5‐methylcytidine methylation

## Abstract

The DNA demethylating therapy with azacitidine (AZA) is a promising therapeutic strategy for elderly patients with acute myeloid leukemia (AML). AZA primarily inhibits DNA methylation, promotes cell differentiation and apoptosis in AML. However, as a cytosine nucleoside analog, AZA also has the potential to be incorporated into RNA molecules. To assess the impact of AZA on RNA m5C methylation during demethylating therapy, we conducted Nanopore direct‐RNA sequencing on samples from three AML patients pre and after demethylating therapy, as well as on HL‐60 cells pretreated with AZA. We performed an integrated analysis of the transcriptome and the m5C methylome, contrasting the states of complete remission with those of active disease (AML). Our results revealed an extensive demethylation effect at the RNA level attributable to AZA and found that mRNA m5C modification may play a pivotal role in the progression of AML. Additionally, *S100P* was identified as a biomarker with significant prognostic implications. We also conducted a conjoint analysis of the transcriptome and the m5C methylome of the full‐length transcripts, uncovering several dysregulated mRNA isoforms. Collectively, our findings indicate that mRNA m5C methylation is implicated during AML progression, and AZA exhibits an overall suppressive effect on this process.

**Abbreviations**
3′UTR: 3′ untranslated region
AML: acute myeloid leukemia
AZA: azacitidine
BM: bone marrow
CDS: coding sequence
CR: complete remission
DEGs: differentially expressed genes
DETs: differentially expressed transcripts
DMSO: dimethyl sulfoxide
GO: gene ontology
GTEx: genotype‐tissue expression
HL‐60: human promyelocytic leukemia cells
m5C: 5‐methylcytosine
PCA: principal component analysis
TCGA: The Cancer Genome Atlas
VEN: venetoclax

## 1 Introduction

Acute myeloid leukemia (AML) represents a malignant transformation of myeloid stem/progenitor cells and is the most prevalent form of acute leukemia in adults [1]. As a genetically diverse disease, AML is characterized by a range of identifiable somatic mutations [2]. Despite advancements in sequencing technologies that have uncovered numerous recurrent genetic abnormalities, the clinical outcomes for AML patients have been less than ideal, prompting an ongoing quest for novel mechanisms and therapeutic approaches [3]. Nanopore direct‐RNA sequencing, a long‐read sequencing technique, offers a unique opportunity to examine both the transcriptome and the methylome changes, thereby illuminating the pathogenesis of AML at the mRNA level [4]. Previous studies have employed several analytical methods for Nanopore, such as xPore [5], CHEUI [6], and Nanocompore [7]. Using these methods, RNA epigenetic analyses, such as N6‐Methyladenosine methylation and polyadenylation, have been conducted on various tumor tissues, including multiple myeloma [5], AML [8], clear cell renal carcinoma [9], and bladder cancer [10].

5‐Methylcytosine (m5C) is a critical epigenetic mark found in both DNA and RNA, where it is distinguished by the addition of a methyl group to the 5th carbon of the cytosine base [11]. In mRNA, m5C is particularly enriched in the 3′ untranslated region (3′UTR), within GC‐rich sequences, and around the start codon. It plays a pivotal role in mRNA stability, translation efficiency, and nuclear export, which are fundamental for the translational regulation and the maintenance of cellular homeostasis [12, 13]. The presence of RNA m5C has also been linked to the development and progression of various cancers, including its influence on oncogenic mRNA stability and expression [14, 15], as well as its regulatory role in cancer hallmarks such as proliferation, invasion, migration [16], and metabolic reprogramming [17]. In the realm of AML, recent studies have underscored the importance of m5C in tumor immune microenvironment formation, prognosis, and drug resistance [18, 19]. Mechanistically, Tet methylcytosine dioxygenase 2 (TET2), an RNA demethylase, has been shown to mediate the demethylation of m5C in mRNA, specifically affecting the stability of tetraspanin 13 (Tspan13) mRNA and contributing to leukemic processes [20].

Azacitidine (AZA), a cytosine analog, is known for its ability to integrate into DNA and inhibit DNA methylation [21]. The combination of AZA and venetoclax (VEN) represents an emerging strategy for the treatment of elderly AML patients [22]. The main therapeutic mechanism of AZA is to inhibit DNA methylation and induce cell differentiation and apoptosis in AML treatment [22]. However, previous research has reported that RNA m5C methylation mediates chromatin organization, AZA response and resistance in leukemia cells [23]. Given its nature as a cytosine nucleoside analog, AZA also has the potential to be integrated into RNA molecules [24]. Whether AZA plus VEN therapy affects mRNA m5C methylation during demethylating therapy in AML and the underlying mechanisms are not yet fully understood.

In our study, we collected bone marrow (BM) samples from three AML patients who achieved complete remission (CR) following AZA/VEN treatment and performed Nanopore direct‐RNA sequencing [8]. Additionally, we treated human promyelocytic leukemia cells (HL‐60) with AZA and conducted similar sequencing to observe the drug effects on leukemia cells. Through comprehensive analysis of the m5C methylome and transcriptome, we delineated the global m5C landscape in AML patients before and after AZA demethylating therapy. We discovered a strong correlation between hypermethylated m5C in AML patients and myeloid cell activation‐related phenotypes. Furthermore, our findings indicate that AZA significantly demethylates RNA in AML patients, potentially through the downregulation of RNA m5C regulators. Finally, we identified *S100P* as a biomarker with aberrant m5C modification and differential gene expression. The analysis of full‐length transcripts identified two mRNA isoforms exhibiting anomalous methylation patterns that were inconsistent with the data from The Cancer Genome Atlas (TCGA) database. This discrepancy suggests that m5C methylation might specifically regulate the expression or function of certain mRNA isoforms. This observation underscores the necessity for further full‐length transcript investigation. Our study sheds new light on the role of AZA in mediating mRNA m5C methylation in AML treatment, offering insights into a novel therapeutic mechanism.

## 2 Methods

### 2.1 Patients and Cells

BM samples were meticulously collected from three AML patients who had achieved CR at Beijing Hospital, following the protocol previously outlined [8]. This study has been approved by the Ethics Committee of Beijing Hospital, and all the participants have signed informed consent.

The HL‐60 cells were cultured in an RPMI 1640 medium supplemented with 10% fetal bovine serum. These cells were subjected to treatment with AZA at an IC50 concentration of 1.86 μM or a control treatment with dimethyl sulfoxide (DMSO), following an established procedure [8]. After a 72‐h treatment, the cells were harvested for subsequent Nanopore sequencing analysis.

### 2.2 Nanopore Direct‐RNA Sequencing

Total RNA was isolated from patient samples and cells using the TRIzol Reagent (Thermo Fisher Scientific) as previously described [8]. The mRNA was purified with the Dynabeads mRNA Purification Kit (Invitrogen). Libraries were prepared using Direct‐RNA Sequencing Kit SQK‐RNA002 (Oxford Nanopore Technologies). Libraries were sequenced on R9.4.1 chips (ONT) using a GridION sequencer.

### 2.3 Analysis of m5C Sites From Nanopore Reads

Upon acquisition of the fasta5 files from Nanopore sequencing, the raw signals were basecalled using Guppy (v3.3.0) (https://community.nanoporetech.com/downloads) to generate the base sequences [25]. The sequence assembly was aligned to human genome reference (GRCh38) using Minimap2 (v2.26) (https://github.com/lh3/minimap2) [26]. Signal data were resquiggled to aligned sequences using Nanopolish (https://nanopolish.readthedocs.io/en/latest/) [27].

To predict m5C modification sites and quantify their probabilities, we employed CHEUI (Methylation (CH3) Estimation Using Ionic Current) (https://github.com/comprna/CHEUI), a sophisticated software that utilizes a two‐stage deep learning method [6]. This approach allows for the detection of m5C across the transcriptome with single‐read and single‐site resolution [6]. The CHEUI model 1 processed the prefiltered signals, calculating the m5C methylation probability at each individual C nucleotide within the reads [6]. Model 2 of CHEUI then proceeded to compute the probability and stoichiometry for m5C at each transcriptomic site [6]. Following this, the CHEUI predict model 1 for m5C was utilized to identify differential RNA m5C modifications between the two groups [6].

Post‐quantification of m5C site probabilities, sites with a m5C probability less than 0.5 were excluded from further analysis. The genes and transcripts corresponding to the detected m5C sites were annotated using BEDTools (v2.26.0), with Gencode (v35) serving as the reference for human genome annotation. The distribution of m5C sites across the human genome was visually represented using the R package RIdeogram (v0.2.2), which provided a comprehensive and illustrative depiction. The sequence logo for m5C motifs was crafted using the R package ggseqlogo (v0.2), offering a graphical summary of the motifs' prevalence. For functional analysis, Gene Ontology (GO) enrichment was performed using the R package clusterProfiler (v4.8.2), which identified biologically relevant categories enriched in differentially methylated genes. The top 10 GO terms were visualized using ggplot2 (v3.4.3), a powerful tool for data visualization.

After obtaining the differential RNA m5C modifications, m5C sites with |difference value | > 0.1, and *p* < 0.1 were considered as differentially methylated in AML and CR samples. For HL‐60 cells, differentially m5C sites were filtered with |difference value | > 0.3, and *p* < 0.1.

### 2.4 Analysis of Gene Expression From Nanopore Reads

The long sequencing reads were mapped to the human genome (GRCh38) using Minimap2. Subsequently, the gene expression matrix was generated by NanoCount (v1.0.0) (https://aslide.github.io/NanoCount) [28]. In the analysis of AML and CR samples, R package DEseq. 2 (v1.40.2) was used to identify differentially expressed genes (DEGs) and differentially expressed transcripts (DETs). For HL‐60 cells, R package Edge R (v3.42.4) was used to determine DEGs and DETs. DEGs or DETs with |logFC| (log2 fold change) > 1 and *p*‐adjust value < 0.05 were considered as differentially expressed. The heatmap of m5C regulators was performed by R package pheatmap (v1.0.12).

The DEGs and DETs were then intersected with the differentially m5C modifications, respectively, to yield a set of commonly regulated genes or mRNA transcripts. The interaction map was generated using R package VennDiagram (v1.7.3).

### 2.5 Public Database Analysis

We used GEPIA 2 (http://gepia2.cancer-pku.cn/#index) to analyze the RNA sequencing data from TCGA and Genotype‐Tissue Expression (GTEx) database. The survival analysis and gene expression analysis were generated from GEPIA 2.

## 3 Results

### 3.1 The Overall m5C Methylation Levels of AML Patients Were Downregulated after Achieving CR

**Figure 1:**
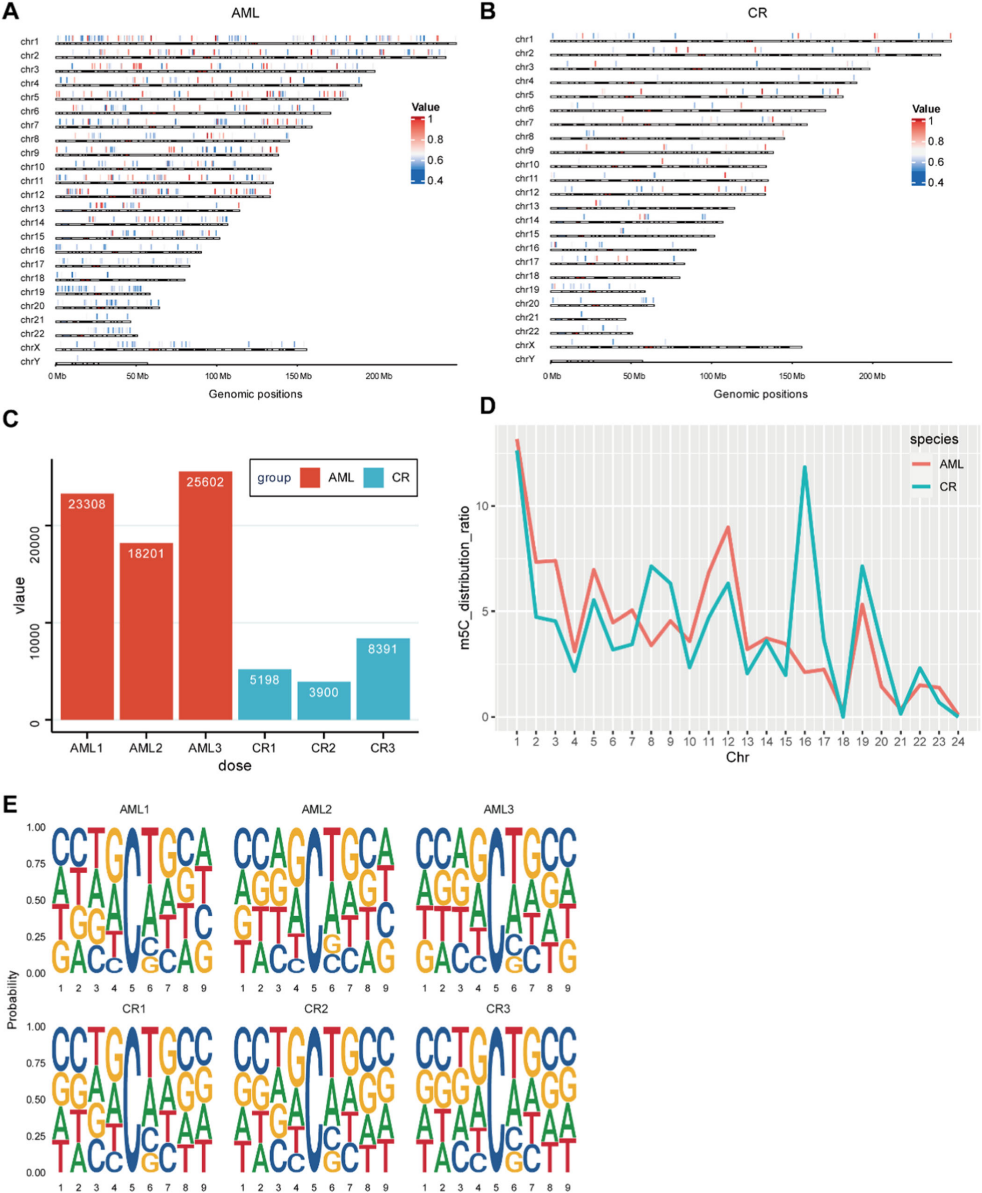
Characteristics of m5C sites in AML and CR BMs. (A, B) The distribution of m5C sites in AML and CR BMs throughout the genome, respectively. The color of scale from red to blue represents the value of m5C level. (C) The number of m5C sites in each group (filtered by m5C value > 0.5). (D) The distribution ratio of m5C sites on each chromosome in AML (red) and CR (blue) BMs. (E) The sequence logos showed the enrichment for m5C motifs in each group.

To assess the impact of AZA on RNA m5C methylation during AML treatment, we conducted Nanopore direct‐RNA sequencing on samples from three AML patients pre and after AZA plus VEN treatment [8] (Figure S1). The m5C profile in AML patients revealed a broad distribution across the genome, with a noticeable absence in the Y‐chromosome region (Figure 1A). Upon achieving CR, a marked decrease was observed in the overall m5C methylation levels (Figure 1B). Concurrently, a significant reduction in the number of m5C sites was observed (Figure 1C). A subtle yet discernible difference in the distribution ratio of m5C sites on each chromosome was noted between the AML and CR groups, particularly on chromosome 16 (Figure 1D). Sequence frequency logo analysis provided further insight, demonstrating an even distribution of the four types of bases surrounding the mRNA m5C sites (Figure 1E).

To elucidate the inhibitory effect of AZA on m5C methylation in leukemia cells, we treated HL‐60 cells with AZA at the IC_50_ and performed Nanopore direct‐RNA sequencing. Our findings indicated that m5C methylation sites were dispersed in both DMSO and AZA‐treated groups (Figure S2A,B). Importantly, AZA treatment led to a significant suppression of the overall m5C methylation levels in HL‐60 cells (Figure S2A–C). This suggests that AZA exerts a profound inhibitory influence on the epigenetic landscape of HL‐60 cells across the entire genome. However, the distribution ratio of m5C sites on each chromosome remained largely consistent between the two groups, with the exception of chromosomes 19 and 21 (Figure S2D). The sequence frequency logo analysis mirrored the earlier findings, showing a uniform distribution of the four bases around the mRNA m5C sites (Figure S2E). Taken together, these findings underscore that the post‐CR patients exhibit a general decrease in m5C methylation. This reduction is likely predominantly attributed to the therapeutic effects of AZA, which reshapes the methylation profile in AML patients and potentially contributes to remission.

### 3.2 Hypermethylated m5C in AML Patients Strongly Related to Myeloid Cell Activation‐Related Phenotypes

Unlike solid tumors, distinguishing between malignant and adjacent nonmalignant tissues in AML, a hematological malignancy, presents unique challenges. Clinically, achieving CR in AML is marked by BM blasts comprising less than 5%, the absence of detectable disease, and the normalization of peripheral blood counts [29]. In this study, the BM tissue samples collected during the AML and CR phases were considered representative of ‘cancerous tissue’ and ‘adjacent noncancerous tissue', respectively.

**Figure 2:**
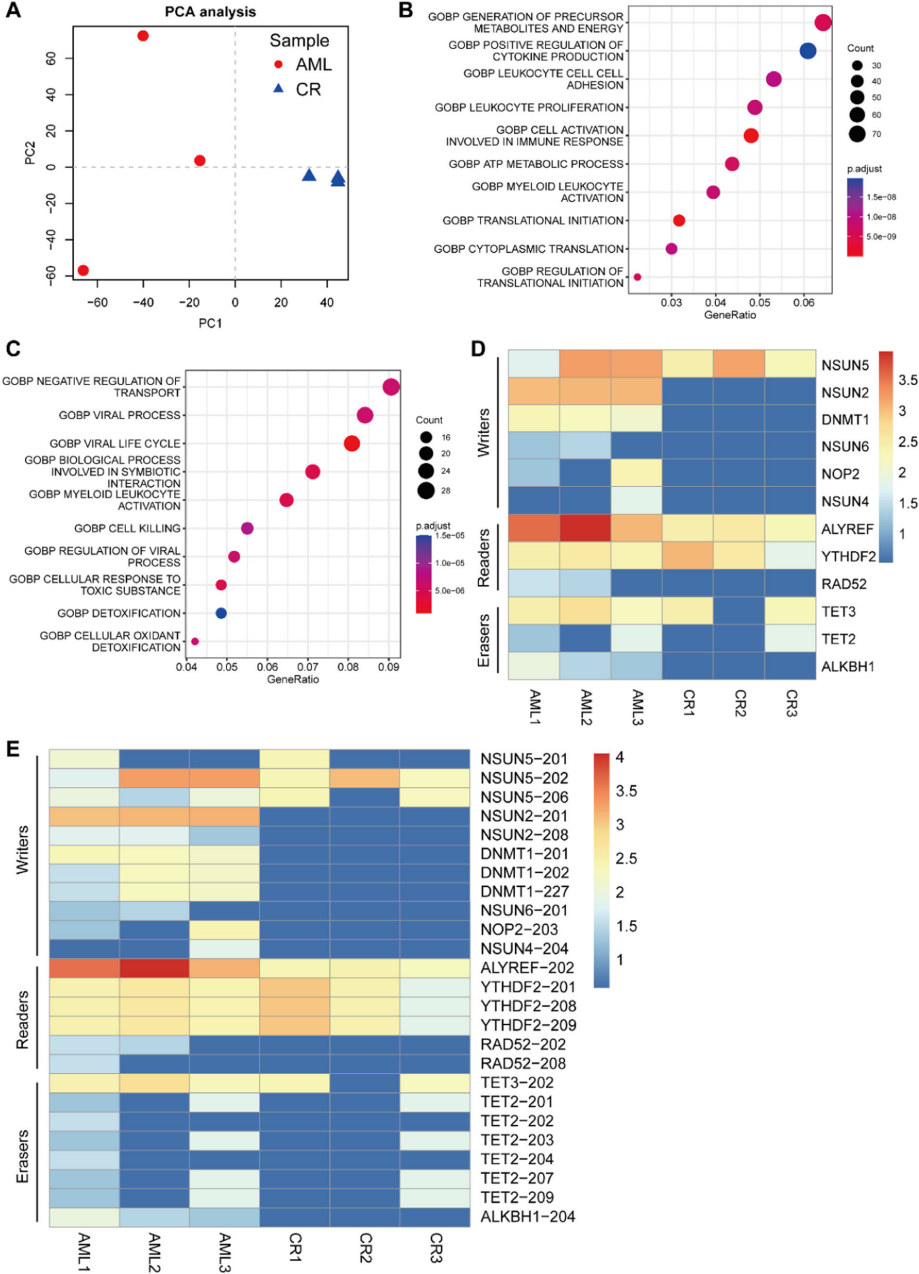
PCA and GO enrichment analysis of hypermethylated m5C. (A) PCA analysis of m5C sites in AML and CR BMs. (B, C) GO enrichment analysis of m5C‐modified mRNAs combined from AML (B) and CR (C) group, respectively. (D) Heatmap for gene expression of RNA m5C regulators. (E) Heatmap for transcript expression of RNA m5C regulators.

Furthermore, we conducted a principal component analysis (PCA) based on all identified m5C sites to delineate the differences between these two groups. PCA revealed a distinct clustering of samples into AML and CR groups. Notably, the AML group exhibited a broader dispersion, whereas the CR group demonstrated a tight clustering, suggesting the divergent roles of m5C methylation in these distinct states (Figure 2A). Next, we combined all m5C‐modified genes in AML and CR groups and performed GO pathway enrichment analysis. The m5C‐enriched genes in the AML group were predominantly linked to myeloid cell activation pathways, such as leukocyte cell adhesion, proliferation, activation, and cytokine production (Figure 2B), suggesting that m5C methylation of these genes may regulate the progression of AML. In contrast, in the CR group, hypermethylation of m5C at genes associated with myeloid cell activation was largely diminished. The m5C‐enriched genes in the CR group were primarily involved in pathways related to cellular stress and damage, including symbiotic interactions, cell killing, responses to toxic substances, detoxification, and cellular oxidant detoxification (Figure 2C). Additionally, the immunosuppressive effects of drug treatments, potentially leading to infections, were reflected in the enrichment of antiviral response pathways (Figure 2C). In summary, these findings indicate that mRNA‐level m5C methylation of genes may play a pivotal role in the progression of AML. A reduction in overall m5C methylation levels posttreatment could serve as a significant biomarker for disease remission. In essence, the “adjacent noncancerous" tissue in AML exhibits a comparatively lower m5C methylation level than the “cancerous” tissue, particularly for genes implicated in disease progression.

To further investigate the underlying factors contributing to the observed reduction in RNA m5C methylation levels following CR, we scrutinized the expression profiles of key RNA m5C regulators. This comprehensive analysis encompassed the “writers,” such as DNA Methyltransferase 1 (DNMT1), and the NOP2/Sun domain (NSUN) RNA methyltransferase, “readers,” such as Aly/REF export factor (ALYREF), YTH N6‐methyladenosine RNA binding protein 2 (YTHDF2), and RAD52 homolog DNA repair protein (RAD52), and “erasers,” such as TET2, tet methylcytosine dioxygenase 3 (TET3), and AlkB homolog 1 histone H2A dioxygenase (ALKBH1) [11]. Our findings indicate a consistent downregulation of these regulators in the CR group, with particularly notable decreases observed among the “writers” and “readers” (Figure 2D).

By leveraging the capabilities of Nanopore direct‐RNA sequencing, which excels in the full‐length transcript detection, we conducted a comparative analysis of isoform‐level between the AML and CR groups among the RNA m5C regulators. The heatmap clearly demonstrated a downregulation of these regulators at the mRNA isoform‐level (Figure 2E). Moreover, we found that the expression levels of some mRNA isoforms were significantly downregulated, while others were not obvious. For instance, among the two NSUN2 isoforms identified, NSUN2‐201 exhibited a marked downregulation in the CR group, whereas the variation in NSUN2‐208 was not statistically significant (Figure 2E). Taken together, these collective results underscore that the expression of RNA m5C regulators is diminished at both the gene and isoform levels in patients who following CR. This insight suggests that the overall reduction in m5C methylation levels may be attributed to the downregulation of RNA m5C regulators, offering a plausible explanation for the epigenetic shifts associated with remission in AML.

### 3.3 The Differentially m5C Analysis Indicates That mRNA m5C Methylation Plays a Broad Regulatory Role in AML

**Figure 3:**
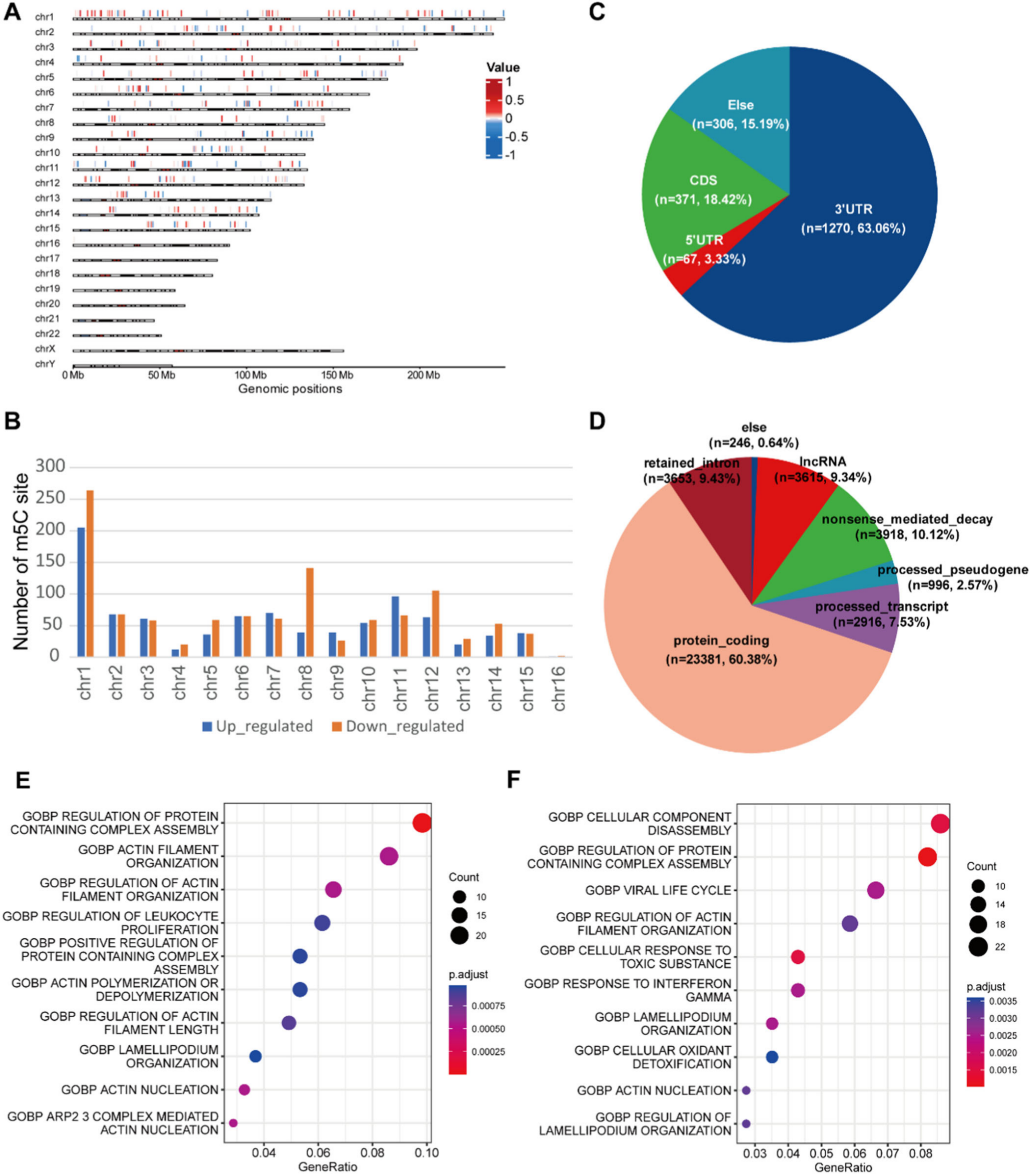
Characteristics of differentially m5C sites between AML and CR BMs. (A) The distribution of differentially m5C sites in CR group compared with AML group throughout the genome. The differentially m5C sites were filtered the with *p* < 0.1 and difference value > 0.1. The red color represents the upregulated m5C sites. The blue color represents the downregulated m5C sites. (B) The number of upregulated (blue) and downregulated (orange) m5C sites on each chromosome. (C) The distribution of differentially m5C sites within functional regions of genes. (D) The types of transcripts exhibiting differentially m5C modifications. (E, F) GO enrichment analysis of upregulated (E) and downregulated (F) m5C‐modified mRNAs, respectively.

To further explore the function of mRNA m5C methylation in AML, we filtered the differentially m5C sites with *p* < 0.1 and difference value > 0.1. A total of 901 upregulated m5C sites and 1113 downregulated m5C sites were obtained in patients achieved CR. These differentially m5C sites were found to be widely distributed across chromosomes 1 through 15, with a slight predominance of downregulated methylation sites over upregulated ones (Figure 3A,B). Next, we detected the distribution pattern of differential m5C sites, and found that m5C sites showed an enrichment in the 3′UTR and coding sequence (CDS) regions (Figure 3C). This finding underscores the pivotal role of m5C in posttranscriptional regulation and translational processes. Moreover, the majority of genes exhibiting differential m5C methylation levels were identified as protein‐coding genes (Figure 3D). This observation further suggests that mRNA m5C methylation may exert a significant influence on the posttranscriptional regulation of protein‐coding genes.

To elucidate the functional implications of differential m5C methylation in AML, we conducted GO enrichment analysis on both the up‐ and downregulated m5C modified genes. The m5C upregulated genes were predominantly enriched in biological processes associated with cytoskeleton dynamics regulation, and the assembly and disassembly of protein complexes (Figure 3E). Conversely, the m5C downregulated genes were primarily associated with processes linked to the assembly and disassembly of cellular components, intracellular environment regulation, and response to external stimuli (Figure 3F). Collectively, these results indicate that m5C methylation plays an extensive role in modulating a wide array of biological processes in AML.

Regarding the impact of AZA on HL‐60 cells, our findings indicated that differential m5C sites were broadly distributed across all chromosomes, with the exception of the Y chromosome (Figure S3A), and a predominance of downregulated methylation sites was observed (Figure S3B). Echoing the patterns observed in patient samples, these differentially m5C sites were predominantly located in the 3′ UTR and CDS regions (Figure S3C), with the majority of affected genes being protein‐coding (Figure S3D). The GO enrichment analysis showed that the m5C upregulated genes were enriched in immune cell regulation, and biological processes related to cellular localization and translation initiation (Figure S3E). In contrast, m5C downregulated genes were associated with biological processes such as cell transport and secretion, leukocyte migration and chemotaxis, and the regulation of autophagy and apoptosis (Figure S3F). These findings suggest that AZA modulates a multitude of cellular functions, including activation, migration, and chemotaxis in leukemia cells.

### 3.4 Conjoint Analysis Pinpoints *S100P* as a Prognostic Biomarker

**Figure 4:**
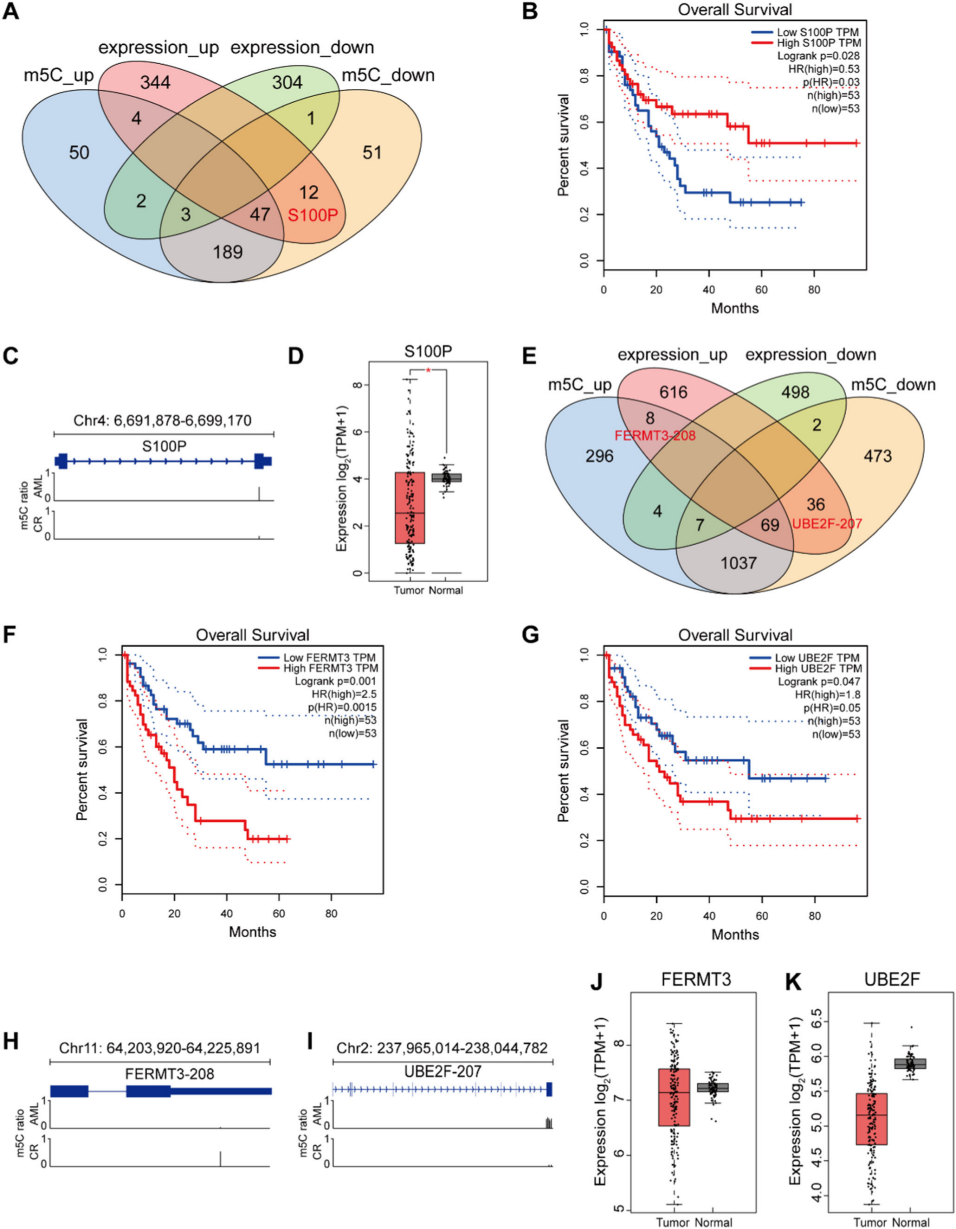
Combine analysis of aberrantly m5C‐modified and DEGs in AML and CR group. (A) The Venn diagram shows the intersection number of aberrantly m5C‐modified and DEGs. *S100P* exhibits hypomethylated m5C and high mRNA expression levels. (B) Overall survival curve of *S100P*. (C) The distribution of m5C site in *S100P*. (D) The gene expression of *S100P* in tumor and normal samples in AML from TCGA and GTEx database. We use log2 (TPM + 1) for log‐scale. *p < 0.05. (E) The Venn diagram shows the intersection number of aberrantly m5C‐modified and DETs. FERMT3‐208 exhibits hypermethylated m5C and high mRNA expression levels. UBE2F‐207 exhibits hypomethylated m5C and high mRNA expression levels. (F, G) Overall survival curve of *FERMT3* (F) and *UBE2F* (G), respectively. (H, I) The distribution of m5C site in FERMT3‐208 (H) and UBE2F‐207 (I), respectively. (J, K) The gene expression of *FERMT3* (J) and *UBE2F* (K) in tumor and normal samples in AML from TCGA and GTEx database, respectively. We use log2 (TPM + 1) for log‐scale. There was no significant difference between two groups.

To uncover the potential of genes as prognostic indicators for the overall survival of AML patients, we conducted an integrated analysis of the transcriptome and m5C methylome. This approach involved a comparative examination of genes that exhibited both aberrant m5C modification and differential expression, pinpointing those with concurrent dysregulation (Figure 4A). A total of 19 genes were identified with abnormal patterns of m5C methylation and RNA expression (Data S1). Notably, the *S100P* gene was identified as being associated with the diagnosis of AML (Figure 4B,C). Intriguingly, *S100P* displayed hypomethylated m5C alongside elevated mRNA expression levels in the CR group (Figure 4A). Further investigation revealed that the expression levels of *S100P* in normal tissues exceeded those found in AML patients, as evidenced by data from both the TCGA and GTEx databases (Figure 4D). This observation is particularly compelling as it suggests a correlation between *S100P* expression and disease status. Synthesizing these findings, our integrated analysis suggests that elevated *S100P* expression is indicative of a favorable prognosis following AZA + VEN treatment in patients with AML. We propose a potential mechanism to explain this association: the reduction in m5C methylation levels may be instrumental in promoting *S100P* expression. This hypothesis is supported by the observed hypomethylation and upregulation of *S100P* in patients who have achieved remission, highlighting the significance of epigenetic modifications in the context of AML treatment and patient outcomes.

### 3.5 The mRNA Isoform‐Level Studies

Given the advantage of Nanopore sequencing in full‐length transcript detection, we conducted an integrated analysis of differentially m5C‐modified transcripts and DETs at the mRNA isoform‐level (Figure 4E). DETs are transcripts that show significant differentially expression level between AML and matched CR groups. A total of 50 dysregulated transcripts were identified (Data S2). Subsequently, utilizing the TCGA database, we conducted a survival analysis on the genes corresponding to these dysregulated transcripts. Our findings revealed that two of the genes represented by these transcripts (FERMT3‐208 and UBE2F‐207) exhibit a significant negative correlation with the overall survival in AML (Figure 4F–I). However, our study revealed that both FERMT3‐208 and UBE2F‐207 exhibited increased expression in the CR group (Figure 4E), which paradoxically contradicts the TCGA findings that higher expression levels are associated with a poor prognosis (Figure 4F,G). Furthermore, no significant difference in gene expression levels was observed between AML samples and normal controls in the TCGA and GTEx databases (Figure 4J,K). These discrepancies suggest that the prognostic value of *FERMT3* and *UBE2F* in AML remains inconclusive and indicate that m5C methylation may exert transcript‐specific regulatory effects, warranting further investigation at the isoform‐level.

After a combined analysis with the data from HL‐60 cells, 25 dysregulated genes (Supplementary Data 3) and 67 dysregulated transcripts (Data S4) were identified, respectively (Figure S4A and S4F). Notably, *NCDN* and *SLC31A2*, characterized by lower m5C methylation levels and higher mRNA expression levels in AZA group, were found to be correlated with AML prognosis (igure S4B–E). We also discovered seven transcripts of interest, including NCDN‐202, SLC31A2‐201, ATP6V0A1‐205, RNASEK‐206, PFKL‐204, GGA1‐201, and UBE2F‐202 (Figure S4F–J). Among them, the m5C methylation sites in NCDN‐202 and SLC31A2‐201 transcript correspond to the same sites within the genes *NCDN* and *SLC31A2*, respectively (Figure S4D,E, Supplementary Figure S5A,B). This suggests that the changes in the m5C methylation sites of these specific transcripts may play a dominant role. Moreover, all transcripts, with the exception of ATP6V0A1‐205, displayed hypomethylated m5C levels after AZA treatment (Figure S5A–G). Interestingly, a transcript of *UBE2F* (UBE2F‐202) exhibited hypomethylated m5C but higher mRNA expression levels in AZA‐treated HL‐60 cells (Figure S4F,G), representing a different mRNA isoform that identified in patient samples (UBE2F‐207) (Figure 4I). This finding suggested that m5C methylation may have regulatory effects on distinct mRNA isoforms. Additionally, we analyzed the expression levels of these genes in AML and normal samples from the TCGA and GTEx databases and found that, aside from the lower expression of *SLC31A2* in normal samples, there were no significant differences in other genes between AML and normal samples (Figure S6A–F). Collectively, these results underscore that m5C methylation on mRNA may serve distinct roles across cellular and tissue contexts, as well as among different mRNA isoforms. This highlights the significance of examining mRNA at both the transcriptome and methylome levels to fully appreciate the nuances of gene regulation in AML.

## 4 Discussion

As a DNA hypomethylating agent, AZA has been extensively used in the treatment of various hematologic and non‐hematologic malignancies. Actually, AZA is capable of integrating into both DNA and RNA, a property that has been less explored [24, 30]. Recently, mRNA m5C has emerged as a novel epigenetic modification, the extent of which remains debatable. Utilizing the innovative Nanopore direct‐RNA sequencing technology, our research has uncovered that AZA influences mRNA m5C methylation in AML patients, suggesting a potential RNA‐mediated therapeutic mechanism. The relationship between mRNA m5C and the progression of AML is not yet fully understood. Our data reveal that mRNA m5C methylation sites are pervasive and exert broad effects in AML patients, suggesting that AZA may reduce overall methylation levels by targeting m5C regulators. Additionally, AZA may exert its demethylating effects by inhibiting the activity of these enzymes, which necessitates further investigation and validation at the proteomic level.

The combination therapy of AZA and VEN operates synergistically to combat leukemic cells by inhibiting DNA methylation and blocking the B‐cell lymphoma‐2 (BCL‐2) protein, respectively. This study positioned the AML and CR groups as analogous to cancerous and adjacent noncancerous tissues, offering a fresh perspective on AML pathogenesis. The Nanopore direct‐RNA sequencing technique, with its long reads and absence of GC bias, provides a more precise methylation analysis, enabling us to uncover a new mechanism by which AZA modulates mRNA m5C methylation during AML treatment.

Through integrated analysis, we identified *S100P*, which exhibited hypomethylated m5C and elevated mRNA expression levels post AZA + VEN treatment. S100P, a member of S100 family of small calcium‐binding proteins, has been implicated in various cancers [31], and has been recognized for its diagnostic value in AML [32, 33]. It is also known to play a role in cellular defense mechanisms and to enhance survival under stress in HL‐60 cells [34]. Our analysis further suggests that the prognostic value of *S100P* in AML treatment may be linked to the reduction of m5C methylation levels, thereby promoting *S100P* expression. Further research is necessary to elucidate the specific mechanisms of *S100P* and m5C in AML.

Nanopore's ability to detect mRNA isoform‐level changes is crucial. The integrated analysis of the methylation profile and transcriptome identified significant isoform‐specific alterations that diverge from TCGA database findings, potentially due to m5C methylation's regulation on gene expression and function. Additionally, we discovered discrepancies in dysregulated genes or transcripts between HL‐60 cells and patient tissues, possibly attributable to the heterogeneous composition of patient BM tissues versus the more uniform cellular components.

However, this study has some limitations. We analyzed only three pairs of BM tissues from AML patients, resulting in a limited sample size. Future studies should incorporate a larger cohort to improve the robustness and generalizability of the findings. Additionally, the molecular mechanisms underlying the relationship between m5C and AML biology remain poorly understood. Future research should aim to validate the identified m5C biomarkers, such as *S100P*, and investigate their potential utility in diagnostic applications. Furthermore, future directions could focus on targeting m5C “erasers” or “writers” to modulate RNA methylation levels, offering a potential therapeutic approach for AML.

In summary, this study delineated the comprehensive m5C landscape in AML patients pre‐ and posttreatment with AZA plus VEN, revealing a substantial demethylation effect of AZA at the mRNA level. This suggests that mRNA m5C modification could play a crucial role in AML progression. Furthermore, this study identifies *S100P* as a biomarker with prognostic significance and underscores the imperative of conducting isoform‐level research.

## Author Contributions

Z.C., L.Z., C.L. and Y.L. analyzed the data. G.S. and Z.Z. carried out the sequencing experiment. Z.C., Y.G. and J.L. performed the calculations. Z.C. wrote the manuscript with input from all authors. F.X., R.F., and C.Z. conceived the study and were in charge of overall direction and planning. All authors have read and agreed to the published version of the manuscript.

## Ethics Statement

The studies involving human participants were reviewed and approved by the Ethics Committee of Beijing Hospital (Approval Number: [2022BJYYEC‐189‐02]).

## Conflicts of Interest

The authors declare no conflicts of interest.

## Supporting information

Supporting information.

Supporting information.

## Data Availability

The data that support the findings of this study are available from the corresponding author upon reasonable request.
