# Supplementary material for: mRNA m5C Alteration in Azacitidine Demethylation Treatment of Acute Myeloid Leukemia
Source: Mol Carcinog. 2024 Dec 17;64(3):502–12. doi: 10.1002/mc.23864 (PMC11814907; doi:10.1002/mc.23864)
Supplement: Supplementary file 1 — Supporting information. [file MC-64-502-s001.docx]

| **m5C_up**  **expression_up** | **m5C_up**  **expression_down** | **m5C_down**  **expression_up** | **m5C_down**  **expression_down** |
| --- | --- | --- | --- |
| MICAL2 | GSTP1 | SMIM1 | DBI |
| PLAAT4 | FOS | YBX1 |  |
| TRABD2A |  | GUK1 |  |
| EIF1B |  | OR2W3 |  |
|  |  | IFITM2 |  |
|  |  | B2M |  |
|  |  | GYPC |  |
|  |  | STRADB |  |
|  |  | S100P |  |
|  |  | HSPB1 |  |
|  |  | DMTN |  |
|  |  | RXRA |  |

**Supplementary Data 1** A total of 19 dysregulated genes with abnormal patterns of m5C methylation and RNA expression between AML and CR group in patient samples.

| **m5C_up**  **expression_up** | **m5C_up**  **expression_down** | **m5C_down**  **expression_up** | **m5C_down**  **expression_down** |
| --- | --- | --- | --- |
| MICAL2-221 | GSTP1-202 | SMIM1-204 | DBI-202 |
| PLAAT4-202 | FOS-201 | YBX1-203 | AC010343.1-201 |
| PLAAT4-201 | RPL14-203 | GUK1-210 |  |
| FERMT3-208 | ANP32B-201 | GUK1-201 |  |
| COA8-203 |  | GUK1-234 |  |
| SNAP23-202 |  | GUK1-208 |  |
| NAGK-205 |  | GUK1-203 |  |
| ARPC1B-222 |  | GUK1-202 |  |
|  |  | OR2W3-201 |  |
|  |  | IFITM2-206 |  |
|  |  | IFITM2-202 |  |
|  |  | IFITM2-205 |  |
|  |  | PFDN5-203 |  |
|  |  | RNF10-203 |  |
|  |  | RNF10-206 |  |
|  |  | ITM2B-201 |  |
|  |  | LGALS3-203 |  |
|  |  | B2M-202 |  |
|  |  | GYPC-201 |  |
|  |  | GYPC-203 |  |
|  |  | GYPC-202 |  |
|  |  | STRADB-202 |  |
|  |  | UBE2F-207 |  |
|  |  | PNRC1-203 |  |
|  |  | HSPB1-203 |  |
|  |  | DMTN-203 |  |
|  |  | DMTN-205 |  |
|  |  | DMTN-206 |  |
|  |  | DMTN-207 |  |
|  |  | DMTN-201 |  |
|  |  | DMTN-202 |  |
|  |  | DMTN-214 |  |
|  |  | SLC25A37-205 |  |
|  |  | BNIP3L-203 |  |
|  |  | BNIP3L-206 |  |
|  |  | RXRA-205 |  |

**Supplementary Data 2** A total of 50 dysregulated transcripts with abnormal patterns of m5C methylation and RNA expression between AML and CR group in patient samples.

| **m5C_up**  **expression_up** | **m5C_up**  **expression_down** | **m5C_down**  **expression_up** | **m5C_down**  **expression_down** |
| --- | --- | --- | --- |
| ZMPSTE24 | CR1 | NCDN | LRRC28 |
| PLPPR2 | SEPTIN9 | HIPK1 | USP10 |
| CTNNB1 | ME2 | RDX | TMC8 |
|  | SULF2 | HIF1A |  |
|  |  | ATXN2L |  |
|  |  | NMT1 |  |
|  |  | NFE2L1 |  |
|  |  | UBE2Z |  |
|  |  | U2AF2 |  |
|  |  | MXD1 |  |
|  |  | IL1RN |  |
|  |  | ATP1B3 |  |
|  |  | H2AC6 |  |
|  |  | MTDH |  |
|  |  | SLC31A2 |  |

**Supplementary Data 3** A total of 25 dysregulated genes with abnormal patterns of m5C methylation and RNA expression between DMSO and AZA group in HL-60 cells.

| **m5C_up**  **expression_up** | **m5C_up**  **expression_down** | **m5C_down**  **expression_up** | **m5C_down**  **expression_down** |
| --- | --- | --- | --- |
| SRRM1-203 | CR1-206 | NCDN-202 | POLR1D-205 |
| ZMPSTE24-201 | ARL6IP4-201 | HIPK1-205 | LAMP1-201 |
| ADK-209 | SEPTIN9-201 | OXA1L-209 | RIN3-212 |
| ATP5F1EP2-201 | ME2-218 | HIF1A-202 | LRRC28-203 |
| TNFRSF12A-202 | CDV3-201 | HIF1A-203 | AC135048.1-202 |
| ATP6V0A1-205 | ADD1-229 | MARK3-211 | USP10-201 |
| PLPPR2-201 | GDI1-207 | PDIA3-201 | RNASEK-206 |
| ERLEC1-202 |  | ATXN2L-202 | PSME3-202 |
| CTNNB1-203 |  | USP10-202 | TMC8-201 |
| ADD1-202 |  | NMT1-211 | HK2-202 |
| GPAT3-208 |  | NFE2L1-201 | HMGB1P5-202 |
| GPAT3-202 |  | UBE2Z-201 | ATP6V1B2-206 |
| CCT5-201 |  | PRKAR1A-214 | DOCK8-216 |
|  |  | SLC16A3-216 |  |
|  |  | U2AF2-202 |  |
|  |  | MXD1-201 |  |
|  |  | IL1RN-203 |  |
|  |  | IL1RN-201 |  |
|  |  | IL1RN-202 |  |
|  |  | UBE2F-202 |  |
|  |  | ADRM1-205 |  |
|  |  | MIR3648-1-201 |  |
|  |  | 5_8S_rRNA.5-201 |  |
|  |  | PFKL-204 |  |
|  |  | GGA1-201 |  |
|  |  | POLR2F-209 |  |
|  |  | ATP1B3-202 |  |
|  |  | MAEA-209 |  |
|  |  | H2AC6-201 |  |
|  |  | DNAJB6-211 |  |
|  |  | MTDH-201 |  |
|  |  | SLC31A2-201 |  |
|  |  | DDX3X-265 |  |
|  |  | DDX3X-260 |  |

**Supplementary Data 4** A total of 67 dysregulated transcripts with abnormal patterns of m5C methylation and RNA expression between DMSO and AZA group in HL-60 cells.
