# Supplementary material for: mRNA m5C Alteration in Azacitidine Demethylation Treatment of Acute Myeloid Leukemia
Source: Mol Carcinog. 2024 Dec 17;64(3):502–12. doi: 10.1002/mc.23864 (PMC11814907; doi:10.1002/mc.23864)
Supplement: Supplementary file 2 — Supporting information. [file MC-64-502-s002.docx]

**Supplementary figure 1** The workflow of the experimental and analytical steps.
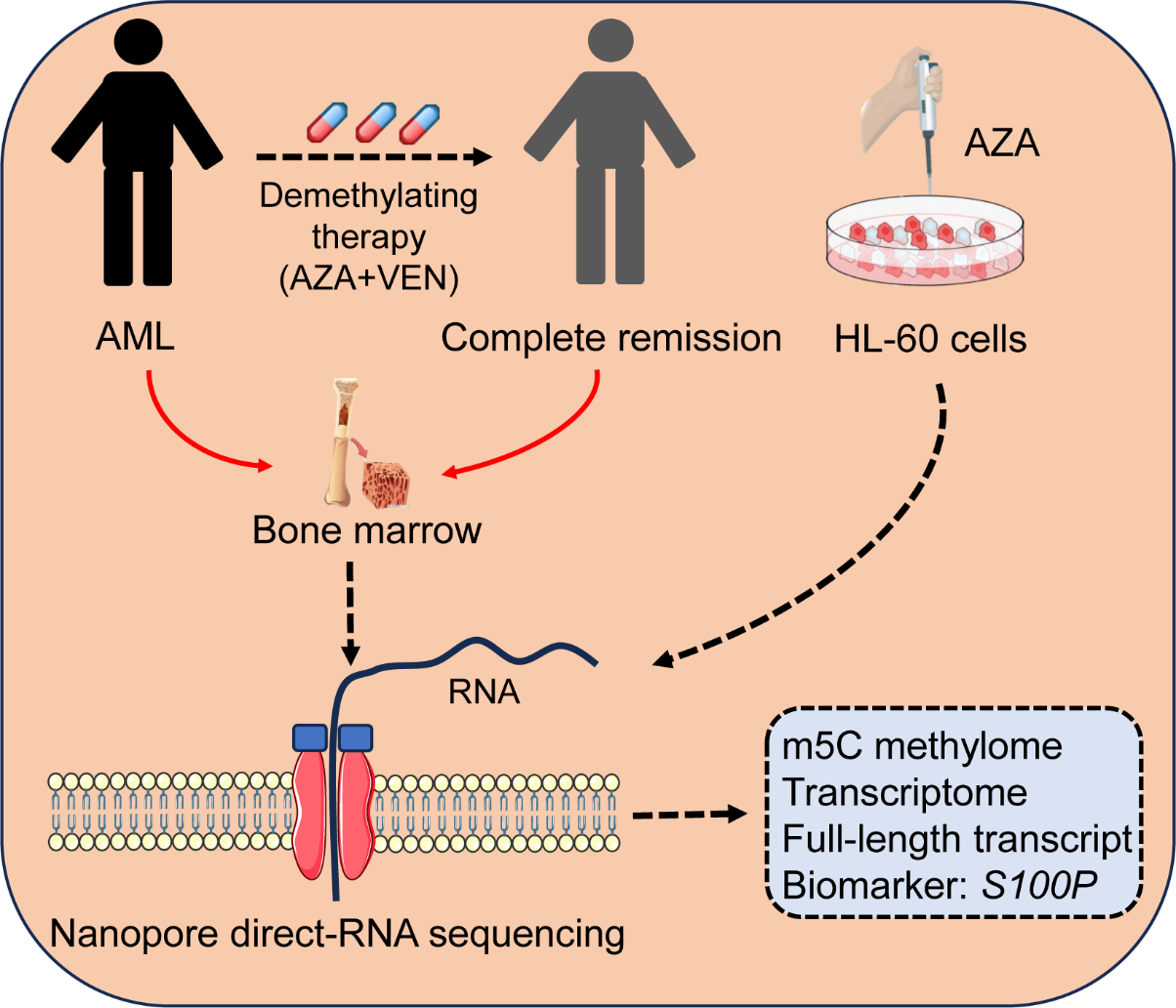


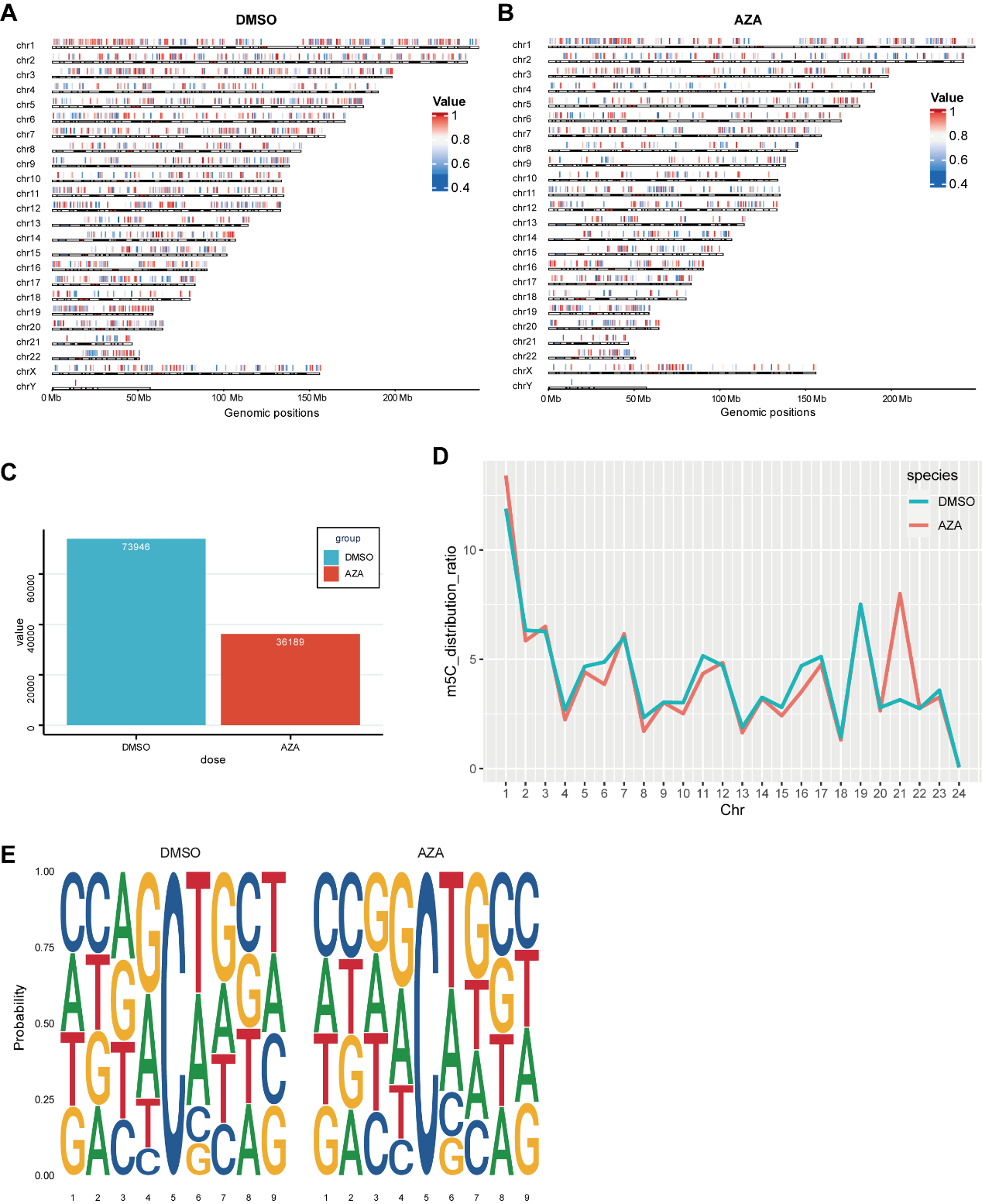
**Supplementary figure 2 Characteristics of m5C sites in HL-60 cells.** A-B. The distribution of m5C sites in DMSO and AZA-treated HL-60 cells throughout the genome, respectively. The color of scale from red to blue represents the value of m5C level. C. The number of m5C sites in each group (filtered by m5C value > 0.5). D. The distribution ratio of m5C sites on each chromosome in DMSO (blue) and AZA (red)-treated HL-60 cells. E. The sequence logos showed the enrichment for m5C motifs in each group.


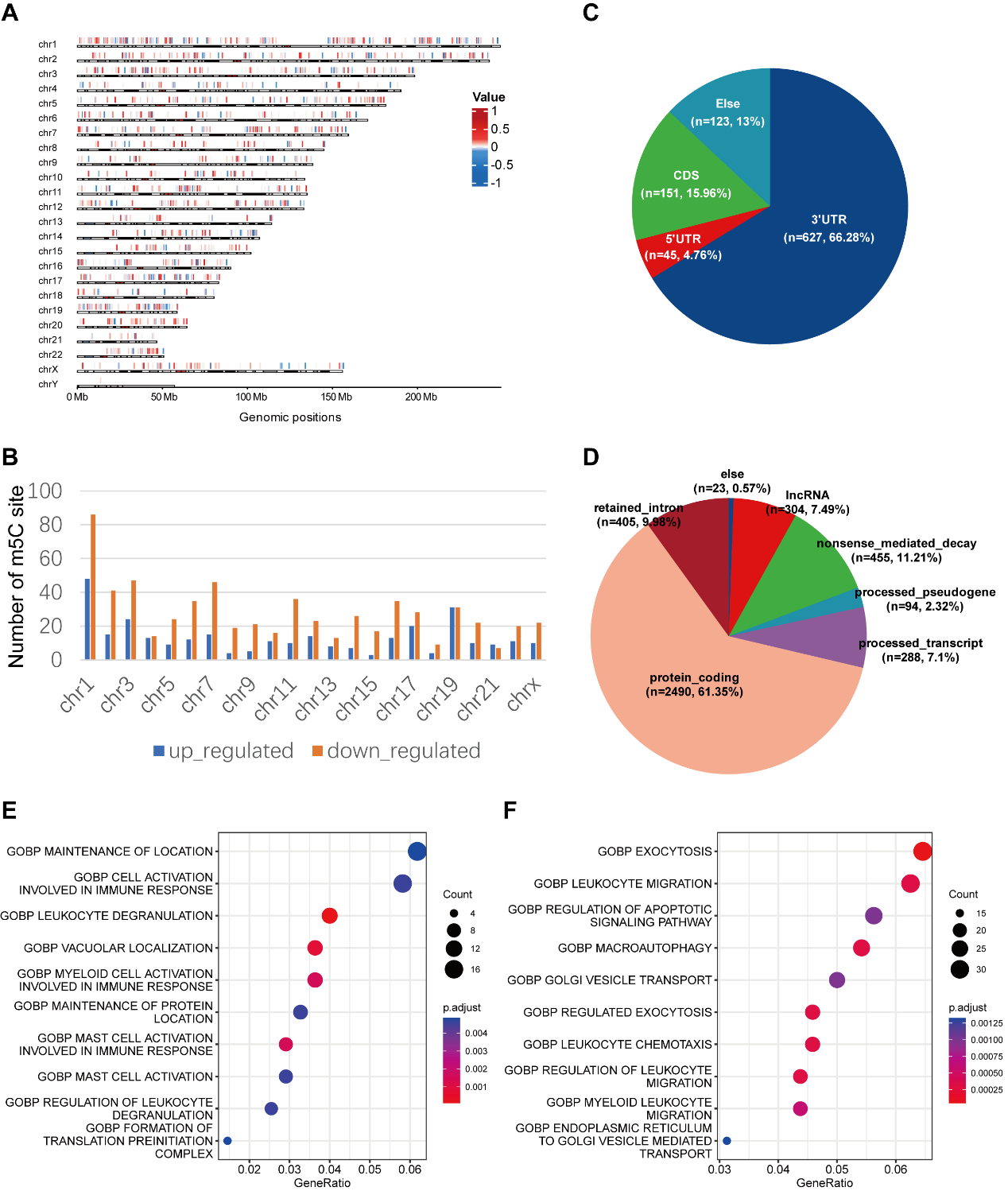


**Supplementary figure 3 Characteristics of differentially m5C sites between DMSO and AZA-treated HL-60 cells.** A. The distribution of differentially m5C sites in AZA group compared with DMSO group throughout the genome. The red color represents the up-regulated m5C sites. The blue color represents the down-regulated m5C sites. B. The number of up-regulated (blue) and down-regulated (orange) m5C sites on each chromosome. C. The distribution of differentially m5C sites within functional regions of genes. D. The types of genes exhibiting differentially m5C modifications. E-F. GO enrichment analysis of up-regulated (E) and down-regulated (F) m5C-modified mRNAs, respectively.


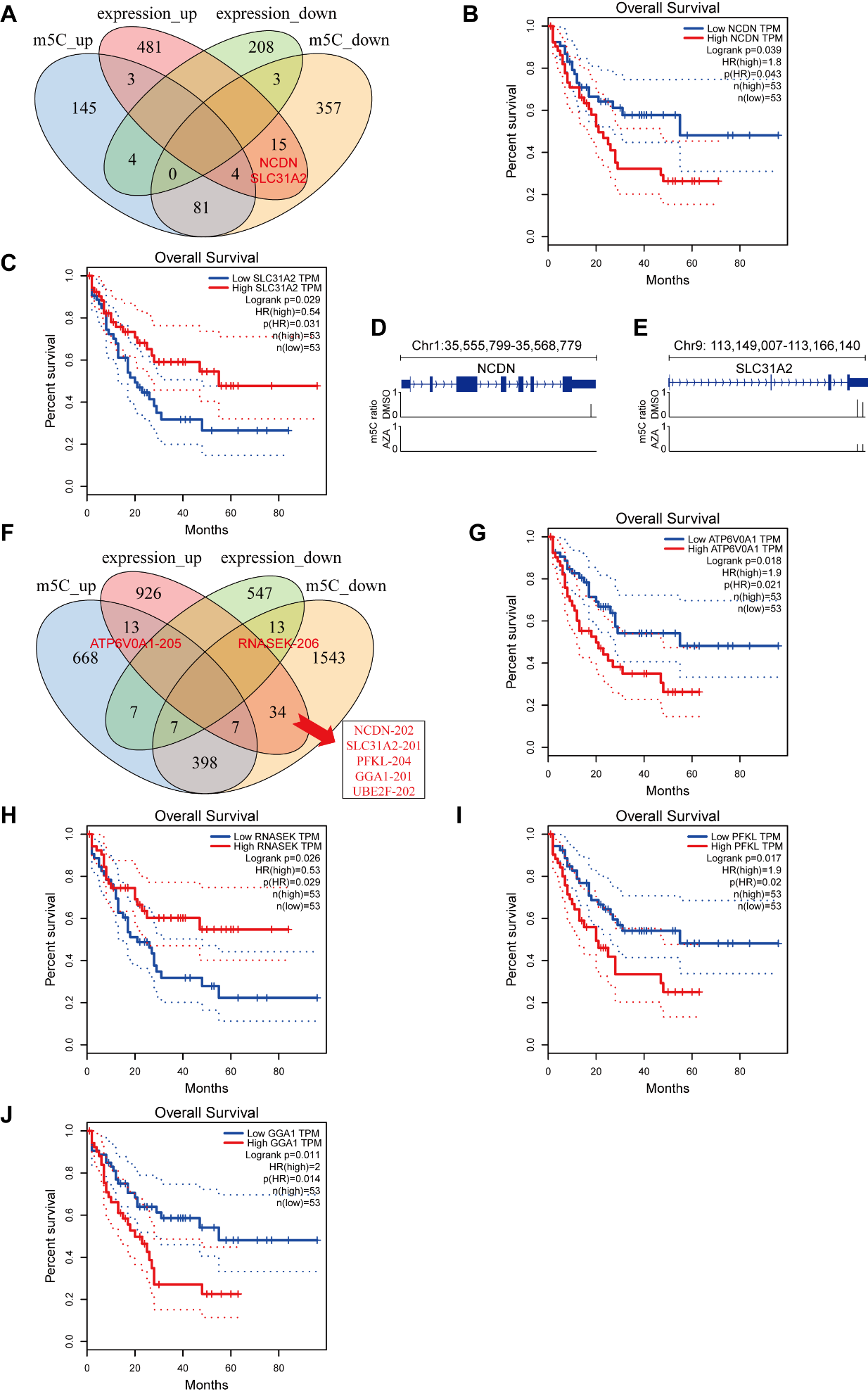


**Supplementary figure 4** **Combine analysis of aberrantly m5C-modified and differentially expressed genes in HL-60 cells.** A. The Venn diagram shows the intersection number of aberrantly m5C-modified and differentially expressed genes. *NCDN* and *SLC31A2* exhibit hypomethylated m5C and high mRNA expression levels. B-C. Overall survival curve of *NCDN* (B) and *SLC31A2* (C), respectively. D-E. The distribution of m5C sites in *NCDN* (D) and *SLC31A2* (E), respectively. F. The Venn diagram shows the intersection number of aberrantly m5C-modified and differentially expressed transcripts. ATP6V0A1-205 exhibits hypermethylated m5C and high mRNA expression levels. RNASEK-206 exhibits hypomethylated m5C and low mRNA expression levels. NCDN-202, SLC31A2-201, PFKL-204, GGA1-201, UBE2F-202, exhibits hypomethylated m5C and high mRNA expression levels. G-J. Overall survival curve of *ATP6V0A1* (G), *RNASEK* (H), *PFKL* (I), and *GGA1* (J), respectively.


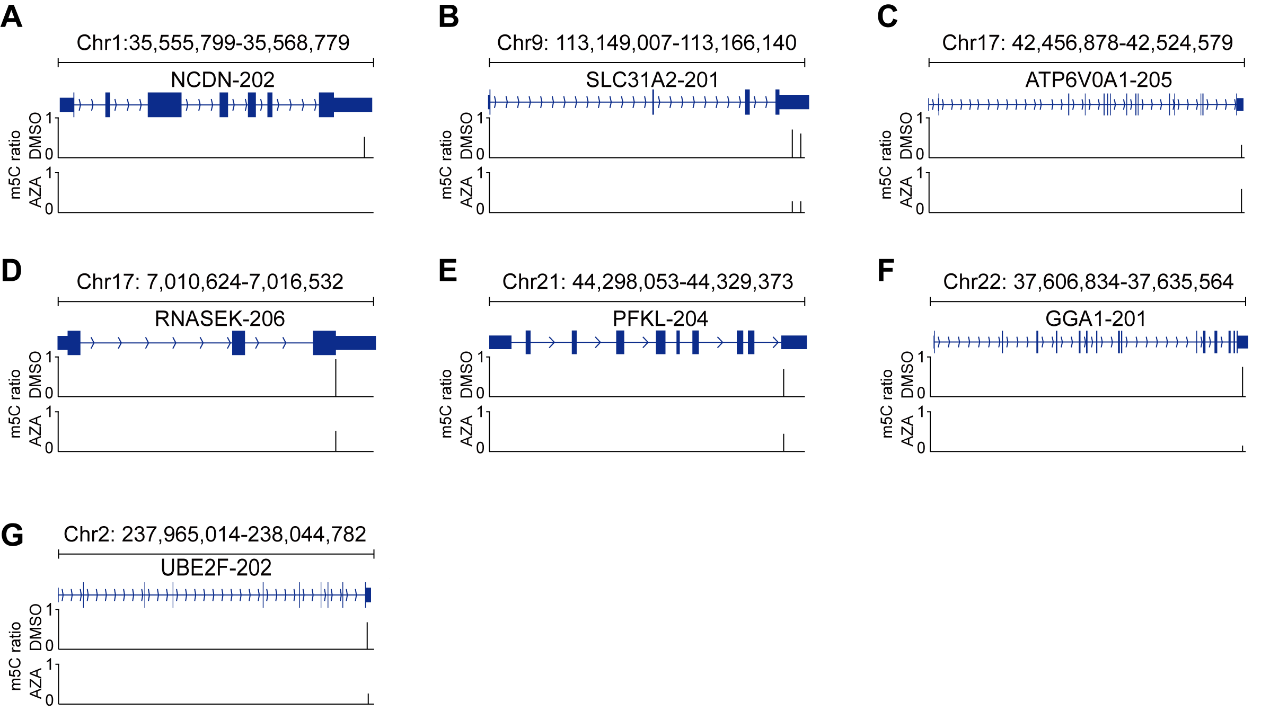


**Supplementary figure 5** A-G. The distribution of m5C sites in NCDN-202 (A), SLC31A2-201 (B), ATP6V0A1-205 (C), RNASEK-206 (D), PFKL-204 (E), GGA1-201 (F), and UBE2F-202 (G), respectively.


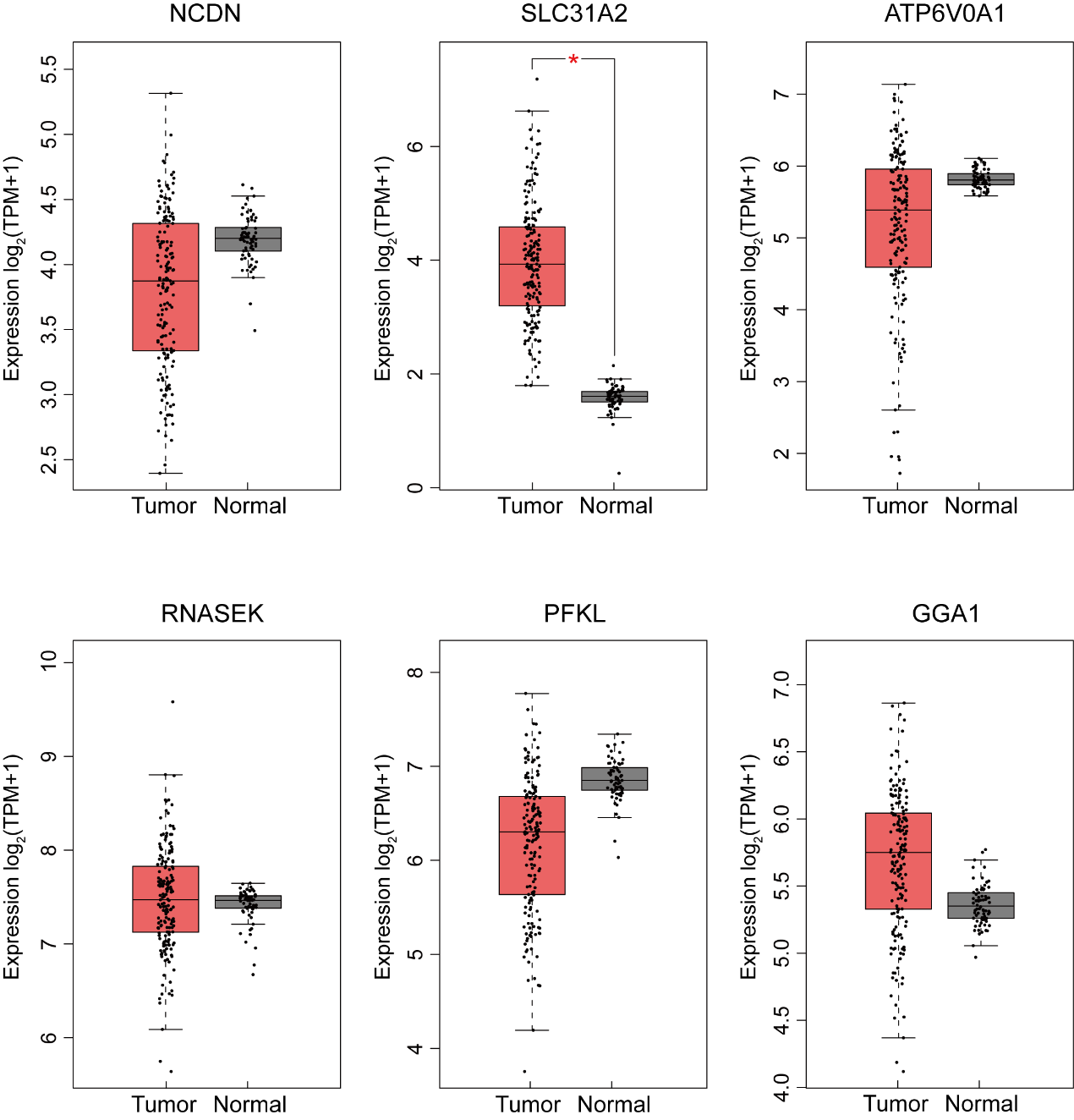


**Supplementary figure 6** The gene expression of *NCDN* (A), *SLC31A2* (B), *ATP6V0A1* (C), *RNASEK* (D), *PFKL* (E), and *GGA1* (F) in tumor and normal samples in AML from TCGA and GTEx database. We use log2(TPM + 1) for log-scale. **P* < 0.05.
